# The Colonization of Rumen Microbiota and Intervention in Pre-Weaned Ruminants

**DOI:** 10.3390/ani13060994

**Published:** 2023-03-09

**Authors:** Kenan Li, Binlin Shi, Renhua Na

**Affiliations:** College of Animal Science, Inner Mongolia Agricultural University, Hohhot 010018, China

**Keywords:** microbiota colonization, pre-weaned ruminant, rumen microbiota-host interaction, nutritional interventions

## Abstract

**Simple Summary:**

In pre-weaned ruminants, the colonization of rumen microbiota is crucial for rumen development and host metabolism. Hence, understanding the colonization process of the rumen microbiota in neonatal ruminants and the factors that affect it may have a positive impact on host health and growth. Rumen microbiota colonizes, and are rapidly established, after birth and constantly interacts with the host. The developing microbial community is more malleable and this may provide an opportunity for nutritional interventions to improve rumen fermentation and increase animal productivity. This paper reviews the latest advances in the colonization of rumen microbiota while providing insights into the most suitable time for manipulating rumen microbial colonization in early life. Furthermore, this manuscript also presents a number of potential factors that influence the establishment of rumen microbiota in early life to optimally enhance rumen development.

**Abstract:**

In pre-weaned ruminants, the microbiota colonizes rapidly in the rumen after birth and constantly interacts with the host to sustain health and metabolism. The developing microbial community is more malleable, so its manipulation may improve ruminant health and productivity as well as may have long-term effects on ruminants. Hence, understanding the process of rumen microbiota establishment is helpful for nutritional interventions of rumen microbiota in pre-weaned ruminants. This paper reviews the latest advances in the colonization of rumen microbiota while providing insights into the most suitable time for manipulating rumen microbial colonization in early life. In addition, different factors that affect rumen microbiota establishment during the pre-weaned ruminants are discussed in the current manuscript. The purpose of this review is to aid in the development of guidelines for manipulating rumen microbiota to improve animal productivity and health.

## 1. Introduction

The rumen has the unique ability to utilize various feeds, which is mainly attributed to the complex, diverse, and non-pathogenic microorganisms inhabiting the rumen, including bacteria, fungi, protozoa, and archaea [1]. These microbes work closely together to break down plant organic matter and provide volatile fatty acids (VFAs) to the ruminants. Thus, ruminants have a special ability to convert roughage into nutrient-rich meat and milk through microbial fermentation.

The world’s population is expected to peak at 9.7 billion in 2050 [2] when demand for meat and milk will increase significantly [1]. As a result, meeting the demand for meat and milk will become a priority for global food security. Researchers have been working for decades on nutritional manipulations of the rumen microbiota to improve fiber utilization or decrease methane production [3]. Nevertheless, the utility of such nutritional manipulation is often inefficient, principally due to redundancy (overlap of physiological capabilities among various microbial taxa) and resilience (capacity to recover its structure following perturbation) within the rumen of adult animals [4]. In newborn ruminants, the developing microbial community is more malleable, so the effects of nutritional manipulation on the rumen microbial community structure may persist for some time after the manipulation is stopped [5]. Consequently, the newborn ruminants’ microbiota provides a suitable opportunity for interventions in such a complicated microbial environment of the rumen.

Previous studies have revealed that the rumen development process could be divided into three phases: the non-rumination phase (from birth to week 3); the transition phase (from week 3 to 8); and the rumination phase (from week 8 onwards) [6,7]. In the process of rumen development, once the rumen microorganisms begin to colonize, the host-associated commensal microbiota is very important for rumen development [8]. To date, there are many studies on the bacterial and archaea establishment process in early life [9,10,11]. Nevertheless, the establishment process of the other rumen microbiota has rarely been reported. This paper focuses on the colonization of rumen microbiota while providing insights into the factors that affect their establishment during the pre-weaned ruminants. In addition, this manuscript will also provide information about the most suitable time for manipulating rumen microbial colonization in early life. The purpose of this review is to aid in the development of guidelines for manipulating rumen microbiota to improve animal productivity and host health.

## 2. Establishment of Rumen Microbiota in Pre-Weaned Ruminants

### 2.1. Establishment of Rumen Bacteria

It is generally believed that the gastrointestinal tract (GIT) of most ruminants is free of microbes at birth [7]. Then, the microbiota from the maternal vaginal, milk, and the surrounding environment rapidly colonize the rumen after birth [6]. However, Bi et al. [12] revealed that a microbiome with low diversity and biomass could be detected in the prenatal gut of lambs, indicating that the prenatal gut harbors a microbiome and that microbial colonization of the fetal gut begins in utero. In contrast, some studies have suggested that microbes detected in utero may be due to DNA present in laboratory reagents and equipment contaminants since the microbiome does not show immune activity or virulence [13,14]. Thus, there is no consensus as to whether the gut microbial community begins colonization in the fetal gut before delivery.

Bacteria essential for mature rumen function can be detected in the rumen of 1-day-old ruminants. For example, Malmuthuge et al. [15] found that *Veillonella*, followed by *Prevotella*, *Bacteroides*, *Eubacterium*, *Streptococcus*, *Acidaminococcus*, *Clostridium*, *Bifidobacterium*, and *Ruminococcus* were predominant (account for 88.7%) in the calf rumen at birth. In addition, the typical rumen bacteria *Eubacterium ruminantium*, *Ruminococcus ruminicola*, *Ruminococcus flavefaciens*, and *Ruminococcus albus*, which can degrade plant polysaccharides, colonize the rumen during the first week of life when calves are fed milk only [15]. The presence of typical rumen bacteria during the first week of life suggests that cellulolytic bacteria colonization occurs in the absence of solid feed. In line with the results of Malmuthuge et al. [15]. Yin et al. [9] found that bacteria responsible for digesting solid feed colonized the rumen before lambs were fed solid feed.

Several studies have found that bacteria colonize and are rapidly established in the rumen in a defined and progressive sequence during the pre-weaning period [7,10,16,17]. Rey et al. [18] found that the bacterial community of calves was established in three successive stages, namely 2 days of age, 3–12 days of age, and 15–83 days of age. Specifically, at 2 days of age, *Proteobacteria* (70%) and *Bacteroidetes* (14%) were the dominant phyla. During postnatal 3 to 12 days, *Proteobacteria* was gradually replaced by *Bacteroidetes* as the main phyla. The most abundant genera were *Bacteroides* (21%), followed by *Prevotella* (11%), *Fusobacterium* (5%), and *Streptococcus* (4%). During postnatal 15 to 83 days, the *Bacteroidetes* phylum was mainly composed of *Prevotella* (42%). During this stage, the composition structure of rumen bacterial flora at the phylum level did not change significantly with time, but only the relative abundance at the genus level still changed accordingly [18]. Similar to the pattern of rumen bacterial colonization in calves, rumen bacterial colonization in lambs is also divided into three consecutive stages, namely 0–3, 10–20, and 20–60 days of age [9]. The *Proteobacteria* were replaced by *Bacteroidetes* as the dominant phylum at the initial colonization process of lambs, the genera *Bacteroides* was the main transitional taxa, and *Prevotella* were the main mature taxa [10,17]. However, there are still some differences in the colonization process of rumen microbes between calves and goat kids. Li et al. [10] and Zhang et al. [17] reported that the colonization process of the rumen bacteria in goat kids can be divided into three stages: the initial phase (0–14 days after birth), transition phase (14–28 days after birth), and relative stable phase (28–56 days after birth). During the initial phase, the genera *Bacillus* and *Lactococcus* were predominant in newborns. As the dominant maternal vaginal flora, the genera *Bacillus* and *Lactococcus* were delivered to the next generation and play a key role in host-immune regulation [19]. However, the colonization of *Bacillus* and *Lactococcus* was transient and it was rapidly replaced by the genera known to readily degrade milk nutrients (e.g., *Bacteroides*). During the transition phase, the genera *Bacteroides*, *Bacteroidales BS11 gut group*, *Alloprevotella*, and *Ruminococcaceae NK4A214 group* were the main transitional taxa. During the relatively stable phase, the genera capable of degrading plant fibers were increased, such as *Prevotella*, *Treponema*, *Ruminococcus*, and the unclassified *Prevotellaceae* were the main mature taxa.

**Figure 1:**
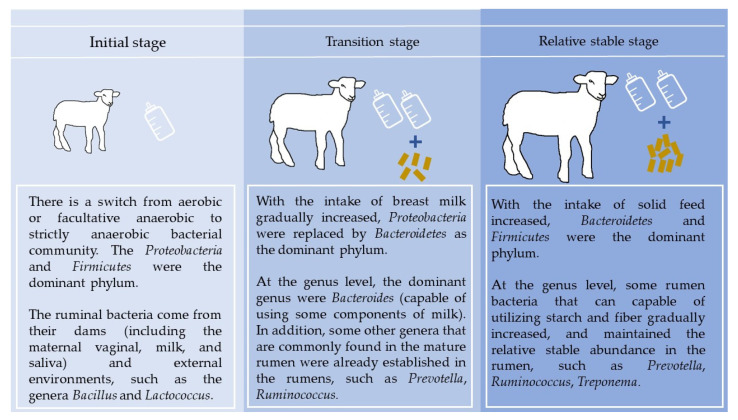
Establishment of rumen bacteria in per-weaned ruminants.

Taken together, the above studies suggest that changes in age may be the main factor affecting the microbial colonization of the rumen. Keeping in view the above studies, the early rumen microbial establishment process can be divided into three stages (Figure 1): (1) In the initial stage, there is a switch from aerobic or facultative anaerobic to strictly anaerobic bacterial community [20]. At the phylum level, the *Proteobacteria* and *Firmicutes* were the dominant phyla [10,18]. The ruminal bacteria come from their dams (including the maternal vaginal, milk, and saliva) and external environments [21], such as the genera *Bacillus* and *Lactococcus*. (2) In the transition stage, with the intake of breast milk gradually increased, *Proteobacteria* were replaced by *Bacteroidetes* as the dominant phylum. At the genus level, *Bacteroides*, capable of using some components of milk, were the dominant genus. In addition, some rumen bacteria that are commonly found in the mature rumen were already established in the rumens, such as *Prevotella* and *Ruminococcus* [17,22]. (3) In the relatively stable stage, with the intake of solid feed increased, *Bacteroidetes* and *Firmicutes* were the dominant phylum. At the genus level, some rumen bacteria that can be capable of utilizing starch and fiber gradually increased, and maintained the relative stable abundance in the rumen, such as *Prevotella*, *Ruminococcus*, and *Treponema* [9,10,18].

### 2.2. Establishment of Rumen Methanogenic Archaea

Typically, archaeal communities contribute to only 3–4% of the rumen microbiota, while methanogenic archaea are a phylogenetically diverse group of archaea [23]. Early studies have found that methanogens begin to colonize the rumen as early as 2–4 days of age in lambs, and the abundance of methanogens reaches adult ruminant levels by 10–14 days of age [24]. Nevertheless, the viability and metabolic activity of methanogens in the first days of life remained unknown. Using real-time quantification PCR (qRT-PCR), the existence of metabolically active methanogens (*Methanobrevibacter mobile, Methanobrevibacter votae*, and *Methanobrevibacter* spp.) were found in the rumen of calves within 20 min after birth [25]. Friedman et al. [26] investigated the metabolic potential and taxonomic composition of the methanogenic archaeal communities across different rumen developmental stages. The results indicate that methanogens in newborn calves have metabolic functions and the activity of methylotrophic methanogens was higher in the first 2 months after birth. In contrast, the activity of hydrogenotrophic methanogens was higher in adult ruminants. However, Malmuthuge et al. [15] discovered that no archaea colonization was detected in calves at birth and the methyl coenzyme M reductase (*mcr*A) gene was observed in weeks 3 and 6 after birth, but not in weeks 1 of calves. Since the *mcr*A gene encodes the α-subunit of methyl coenzyme M reductase, which catalyzes the last step of methanogenesis [27], suggesting that the methanogens had no metabolic activity in weeks 1 of calves. Therefore, studies are still needed on methanogen’s initial colonization and its actual metabolic functionality in the newborn ruminant. It has been reported that the rumen bacterial composition of the goat changes remarkably with age. In contrast, archaeal communities respond less to age [23]. The dominant phyla in the rumen fluid of goats in different age groups were *Euryarchaeota* (82%) and *Thaumarchaeota* (15%) [23]. The abundance of *Euryarchaeota* increased gradually from 1 to 15 days of age and then tended to be stable, while the abundance of *Thaumarchaeota* decreased gradually from birth to 15 days of age and then tended to be stable [23]. Other studies have suggested that feeding system and age were the two main factors affecting methanogen diversity [11,28]. For example, Jiao [28] explored the variation in feeding system (supplemental feeding versus grazing) and the changes related to age in rumen microbial colonization, the results showed that microbial colonization in the rumen is achieved at 1 month and methanogens alpha diversity indices were less for supplemental feeding versus grazing; in addition, within the genus, *Methanobrevibacter*, *Methanobrevibacter ruminantium* clade, and *Methanobrevibacter gottschalkii* clade surged in abundance after the solid feed was offered, while *Methanobrevibacter acididurans* clade only dominated on 70 days after birth. *Methanimicrococcus, Methanomicrobium*, and *Methanosphaera* are minor genera while Rumen cluster C is dominant [28]. Wang et al. [11] used qRT-PCR and 16S rRNA sequencing to study the initial colonization and subsequent changes of metabolically active methanogens in the solid-attached, liquid-associated, protozoal-associated, and epithelial-associated rumen fractions during rumen development from 1 to 60 d after birth in goats, and the results showed that the Chao 1 index increased in an age-dependent manner only in the rumen epithelium-associated methanogens. Methanogens colonized in the rumen liquid-associated and epithelium-associated on the first day of life, and the methanogens densities in four fractions stabilized as the starter diets were introduced. Furthermore, the rumen solid attached accommodated the most methanogens, while the lowest density of methanogens was observed in rumen epithelium-associated. Data obtained from the 16S rRNA sequencing indicated that *Methanobrevibacter*, *Candidatus Methanomethylophilus*, and *Methanosphaera* were basically the top three genera in the four fractions and together represented from 89.8% to 98.3% of total methanogens.

### 2.3. Establishment of Rumen Fungi

It is well known that anaerobic fungi have a critical role in the degradation of plant fiber, primarily due to the enzymes produced by fungi used to degrade plant structural polymers. Moreover, their rhizoids can penetrate plant structural barriers [1]. Compared to bacterial communities, the colonization of rumen fungi seems to appear later [7,29]. For example, the colonization of anaerobic fungi began within 8–10 days after birth in ruminants [29]. However, recent studies have found that the rumen fungal community was rapidly established after the birth of the lambs, and the process of the fungi establishment in the rumen of lambs can be divided into three phases: the initial phase, the transition phase, and the relatively stable phase [17,30]. A study of ruminal fungal community colonization in goats suggested that during the initial colonization of anaerobic fungi before 14 days of age, the *Ascomycota* phylum was the main dominant flora, but after 14 days of age, the *Neocallimastigomycota* phylum gradually became the dominant flora. At the genus level, the abundance of *Aspergillus* reached 47% on d 0, but then it dropped from d 3 to 14, and the abundance of *Neocallimastix*_*sp*, *Orpinomyces*_*sp*, and *Caecomyces* are the predominant fungal genera after d14 [17]. Another study showed that the *Ascomycota* phylum dominated on d 0 and 7, while the *Neocallimastigomycota* phylum dominated on d 28, 42, and 70. Moreover, *Arthrinium*, *Aspergillus*, *Boeremia*, *Candida*, *Neurospora*, and *Purpureocillium* genera dominated on d 0, while the *Candida* genus dominated on d 7 [28].

### 2.4. Establishment of Rumen Protozoa

The rumen is dominated by bacteria, and protozoa account for about 20% or up to 50% of the total rumen microbial population [1]. The main protozoa are ciliates and flagellates [31]. Flagellates mainly exist in the rumen of newborn ruminants. With the increase of age, the number of flagellates gradually decreases and ciliates dominate the protozoa group in the rumen after adulthood. The colonization of protozoa began within 15 days after birth, with small *Entodinia* colonizing before large *Endomorphs* and *Holotrich* protozoa [32,33]. However, if neonatal ruminants are rapidly separated from other ruminants after birth, no protozoa are established in the rumen. This is due to rumen protozoa can only be transmitted from animal to animal through saliva [34].

### 2.5. Rumen Epithelial Microbiota

The rumen epithelial microbiota, defined as the epimural microbiota, is the tissue-attached microorganism of the rumen epithelium. Recent studies have reported that the epimural community is more diverse than the content-associated microbial community [35] and diet can change its composition [36]. In addition, the changes in the epimural bacterial population are associated with host gene expression [37] and ruminal acidosis [38]. The bacterial community is the main member of the epimural microbiota, and the epimural bacterial community has a greater variation in richness among individual ruminants compared to rumen content- and fluid-associated bacteria [39,40,41]. Through a meta-analysis, a study showed that the genera *Campylobacter*, *Prevotella*, *Christensenellaceae R-7 group*, *Butyrivibrio*, and the uncultured genus of the *family Neisseriaceae* being dominant, and the genera *Methanobrevibacter* and *Methanomethylophilaceae* were also identified as the core epimural archaeal taxa [41]. Although several studies have been reported on the diversity and relative abundance of epimural microbial communities [41,42], further studies are needed to determine the establishment process of epimural microbial communities. Moreover, although the epimural microbiota is less than 1% of the total rumen microbiota and has a low contribution to VFA production [43], they are the ones likely to interact directly with the host [44], suggesting that the epimural microbiota plays an important role in rumen epithelial function [45]. To date, the understanding of the epimural microbial community is very limited, although the functions of the epimural microbiota are speculated to be involved in oxygen scavenging, urea recycling, and tissue recycling [45,46]. Therefore, future studies are essential to identify the role of epimural microbiota in rumen function.

### 2.6. The Window of Time for Manipulating Rumen Microbial Colonization in Early Life

There are many studies on the manipulation of rumen microbial colonization in the early stage of life, however, further confirmation of whether nutritional interventions in early life have long-term effects on adult ruminants is still needed. In addition to the potential manipulation strategies described above, it is also important to grasp the most sensitive time window for intervention. The bacterial communities were unstable and more malleable before 20 days of age until weaning (60 days after birth). Therefore, Yin et al. [9] suggested that the first 20 days of life may be the best opportunity for interventions. Different from Yin et al. [9], Li et al. [10] suggested that probiotic intervention during the weaning transition (6–8 weeks) is the best opportunity to manipulate microbiota community composition. As mentioned earlier, the colonization of bacterial, archaeal, and fungal communities occurs before the introduction of a solid diet, and the main microbial communities commonly found in the mature rumen are established in the rumen of young ruminants at 1 week of age [15,22]. Therefore, we suggest that the most suitable time for microbiota manipulation could be right after birth, which agrees with Dias et al. [22] and Abecia et al. [47]. However, the most suitable time for microbiota manipulation and the lasting effects of early nutritional intervention on enhancing rumen fermentation needs to be studied further.

## 3. Factors That Influence Early Life Rumen Microbiota Colonization

Dietary changes, host genotype, age, host immunity, rumen microbial transplantation, and other factors all affect the colonization of rumen microorganisms in young ruminants (Table 1). The effect of the host age on the rumen microbiota has already been discussed.

### 3.1. Dietary Changes

As a substrate for rumen microbial fermentation, the nutrient composition and physical characteristics of the diet directly affect microbial colonization. Dias et al. [22] investigated the effect of a pre-weaned diet (milk versus milk plus starter feeds) on the rumen microbiota of dairy calves, and the results showed that the inclusion of starter feeds facilitated the bacteria, capable of utilizing carbohydrates in the rumen (e.g., *Succinivribrio*, *Sharpea*, and *Megasphaera*); the milk-fed group showed the bacteria (e.g., *Parabacteroides*, *Bacteroides*, and *Lactobacillus*) known to degrade milk nutrients were dominant in the rumen. Recent studies have found that feeding neonatal ruminants with only concentrate can easily cause symptoms such as decreased rumen pH, agglutination of rumen nipples, and incomplete keratinization while adding hay based on concentrate can increase rumen pH, increased rumen volume, and maintaining normal morphology of rumen nipples [54,55,56]. During this process of adding hay based on concentrate, the pre-weaning and post-weaning rumen microbiota were similar, and the fluctuations in bacterial abundance between pre- and post-weaning periods were reduced, promoting the adaptation of neonatal ruminants to the post-weaning diet and reducing weaning stress [48]. This provides a clue for strategies to improve rumen function by manipulating the rumen microbiota in early life. The addition of concentrate or concentrate plus alfalfa pellets to the feed of lambs from 20 to 60 days of age improves the abundance of cellulolytic bacteria and proteolytic bacteria in the rumen content, *Ruminococcus unclassified* drives the rumen epithelium transcriptome and signaling pathways in cell metabolism, and subsequently promote the development of the rumen epithelium, and may be an important genus in influencing lambs’ rumen development [57].

The rumen epithelium is a unique site of interaction between the rumen microbial metabolism and the host [58]. A growing body of research suggests that rumen epithelium development is influenced by lifelong metabolic communication between the rumen microbiota and the host, which develops and changes with diet [8,59]. Therefore, many studies have explored the potential mechanisms of microbiome-host interactions in stimulating ruminal epithelium development through different dietary niches. Wu et al. [60] investigated the diet-rumen microbiota-host interaction in pre-weaned yak calves, and the 16S rRNA sequencing results indicated that butyrate-producing genera were enhanced by the addition of the calf starter or alfalfa hay; transcriptomic results have revealed that alfalfa hay or the starter supplementation upregulated the PIK3-Akt signaling pathway that regulates the development of the ruminal epithelium; the correlation analysis showed that several altered genera (*Christensenella*, *Sphingomonas*, *Vulcaniibacterium*, *shigella*, *Kandleria*, *Escherichia Aquabacterium*, and *Limnobacter*) were positively correlated with butyrate production, and all of them were increased by the starter supplementation. These findings suggest that diet-rumen microbiota-host interactions stimulate the development of rumen epithelium in pre-weaned calves. Lin et al. [59] revealed the mechanism by which starter feeding promotes rumen epithelial development in lambs from the perspective of rumen microbiota-host interaction. The 16S rRNA and 18S rRNA sequencing results showed that the abundance of acetate-producing *Mitsuokella* spp., lactate-producing *Sharpea* spp., lactate-utilizing *Megasphaera* spp., and *Entodinium* spp. were enriched in the rumen microbial community in the starter-feed group; metagenomic results indicated that starter feeding significantly increased the GH13 encoding α-amylase; transcriptome results showed that gene expression of *MAPK1*, *PIK3CB*, *TNFSF10*, *ITGA6*, *SNAI2*, *SAV1*, and *DLG*, which are associated with growth-associated signal pathways, were significantly up-regulated, while gene expression of *BAD*, which is involved in the apoptotic process, was down-regulated; correlation analysis revealed a high correlation between the expressions of these genes and acetate and butyrate concentrations. The data suggest that the increased acetate and butyrate production by the microbiota mediates the expression of growth-related genes in the growth-related signaling pathway in the ruminal epithelium. Overall, these results suggest that the diets not only induce changes in the colonization process of rumen microorganisms but also affect the metabolic function and interaction patterns of rumen microorganisms through diet-rumen microbiota-host interactions.

### 3.2. Host Genotype

During rumen development, changes in rumen structure and physiological properties are associated with the rumen microbiota [61]. Microbial colonization can cause a range of physiological changes in the rumen of newborn ruminants. On the contrary, the host’s physiological changes also cause a series of changes in the microbial composition of the rumen [1]. However, there is no conclusive understanding of whether microbial community alterations lead to functional changes in the host or whether these changes are the result of changes in the host physiology [8]. Pan et al. [62] investigated the rumen transcriptomic and metagenomic data to tease the developmental reprogramming of rumen functions, microbiota colonization, and their functional interactions in seven-time points from postnatal goats. The results showed that both the temporal dynamics of the rumen transcription profile and the microbial metagenome profile exhibit two distinct phases during pre-weaning rumen development; transcriptional profiles of the rumen were divided into the immune-related response phase (d 1–14 after birth) and nutrient-related metabolism phase (d 21–56 after birth); microbial metagenomic profiles of the rumen were divided into bacteriocin biosynthesis phase (d 7–28 after birth) and glycolysis/gluconeogenesis activity phase (d 42–56 after birth); the development shift in the rumen transcriptome (on d 21) was earlier than the feed stimulus (on d 25) and the shift of the rumen microbiome (on d 42). In addition, two newly evolved genes (*LYZ1* and *DEFB1*) showed antibacterial activity against gram-positive bacteria. Taken together, these results suggest that first-stage rumen development is more likely to be a programmed process rather than a result of diet and microbial stimulation and that the newly evolved genes may have specific functions in regulating the composition of the microbiota [62,63]. A study of high- and low-efficiency Holstein cows found that after a near total exchange of rumen contents between the cows, the host restored the rumen bacterial community to its pre-exchange state, suggesting that the rumen microbial community is to some extent controlled by the host genes [64]. In addition to the host genetics, differences in rumen microbiota composition may be due to other factors, including higher cesarean delivery rates in some breeds of cattle, such as Belgian Blue cattle [65]. Moreover, the physiology of the rumen also likely plays an influential role in the rumen microbiota. Henderson et al. [66] identified diet as a major driver of rumen microbiota composition, but they also found some differences between certain bacterial communities in different ruminants, such as the higher abundance of unclassified *Veillonellaceae* in sheep, deer, and camelids compared to cattle, which may be related to differences in the rumen and camelid foregut size, physiology, and feeding frequencies, thus explaining the host specificity of the rumen microbiota. In the early stages of rumen development, there is significant heterogeneity in the composition of the rumen microbial community, and rumen development significantly affects the diversity of rumen bacteria. This is mainly due to substantial changes in the anatomical structure of the rumen, followed by changes in physiological and metabolic functions [67]. For example, the VFA produced by the rumen microbiota affects the size and shape of the rumen papillae, and these rumen papillae structures alter microbial colonization as they provide a niche environment for a particular rumen microbiota [68]. There is increasing evidence that host genetics, age, early life events, and rumen physiology have important effects on the microbial community in the rumen. Nonetheless, our current understanding of the host effect on the establishment of rumen microbiota is still incomplete.

### 3.3. Host Immunity

Due to the nature of fermentation and the constant exposure of the rumen epithelium to microorganisms, physiological factors in the rumen may differ from those in other parts of the GIT. The rumen epithelium consists of up to 15 cell layers, which limits permeability to large molecules, and the rumen epithelium has no organized lymphoid tissue. As stated by Yáñez-Ruiz et al. [7], the microbial equilibrium in the rumen is achieved through a combination of the immunoglobulins, IgG and IgA in saliva, Toll-like receptors (TLRs), genetically encoded pattern recognition receptors, peptidoglycan recognition proteins, and antimicrobial peptides defensins. Newborn ruminants have no immunity at birth, and the immunoglobulins, IgG, IgA, and IgM in colostrum can induce immunity and modulate the intestinal bacterial community composition, maintaining a balance between the intestinal microbiota and the host [7]. Moreover, the colonization of microbiota plays a key role in the development of host innate immunity [69]. Pan et al. [62] revealed that the host immune regulation could be activated after the microbiota colonization on d 1, the bacteriocin produced by rumen microbiota is crucial for the rumen immune response and subsequent microbiota colonization, and the expressions of some immune-related genes (*CEBPE*, *SOCS1*, *SOCS3*, *CCDC3*, and *S100A9*) in the rumen have a lasting effect on the establishment of microbiota. Secreted immunoglobulin A, which favors symbiotic bacteria in the gut [70], has been demonstrated to coat rumen bacteria [71] and regulate the host’s recognition of certain microbial species.

Members of the *TLRs* gene family are important regulatory elements of the rumen epithelium, and under normal physiological conditions, they may respond to changes in the structure of the rumen microbial community and make a corresponding regulatory response to maintain the immune tolerance of the rumen epithelium to symbiotic microorganisms [72]. In monogastric animals, some members of the *TLRs* gene family can inhibit the activation of epithelial NF-*k*B (nuclear factor-kappa B) inflammatory pathway by binding to specific symbiotic secreted proteins, and thus play an important regulatory role in the maintenance of digestive tract epithelial immune tolerance [73]. Shen et al. [74] found that non-fiber carbohydrates (NFC) induced the expansion of commensal bacteria (*Verrucomicrobia subdivision* 5), and promoted the immune tolerance of rumen epithelium by upregulation of *TLR10* expression. In addition, Chen et al. [37] showed that, compared with acidosis-sensitive cattle, acidosis-resistant cattle had different epimural bacteria diversity and higher expressions of *TLR2* and *TLR4* in the rumen epithelium, suggesting that the susceptibility to subacute rumen acidosis may be related to the immune activity of the epithelial tissue, which is induced by the epimural microbiota. Therefore, the epimural microbiota plays a critical role in ruminal immune function through direct interactions with the host [45]. However, there has been little research on the immune system’s response to different microbial colonization patterns. Therefore, further studies are needed to illustrate the impact of different microbial colonization patterns on the immune system and the long-term effects.

### 3.4. Rumen Microbial Transplantation, Probiotics, and Other Factors

Rumen microbial transplantation is an effective method for reconstructing the structure of the rumen microbiota. As carriers of mature rumen function, adult rumen microorganisms are a donor source for microbial transplantation in young ruminants. Compared to specific microbial preparations, rumen microbial transplantation may accelerate the colonization process of rumen microorganisms and may even affect the host’s immune function. Belanche et al. [75] showed that inoculation of neonatal goats with adult goat rumen fluid could facilitate protozoa colonization and promote forage intake, VFA production, and nutrient absorption during the pre-weaning period. The researchers used young lambs as models, rumen fluid transplantation from adult sheep was performed orally before and during weaning, and it has been found that inoculation with fresh rumen fluid promotes bacterial colonization of lambs, such as *Succiniclasticum*, *Prevotella*, and *Proteobacteria S24-7*; rumen fluid transplantation before weaning promoted more microbial colonization than rumen fluid transplantation during weaning [52]. Feeding lyophilized rumen fluid to lambs changed the composition of *Selemonas ruminantium* and *Megasphaera elsdenii*, and reduced the relative abundance of *Ruminococcus flavescens* [76]. The studies indicate the importance of early intervention in rumen microflora. In addition, probiotic supplementation before weaning ruminants can also alter the process of rumen microbial colonization. For instance, when fibrolytic bacteria isolated from the rumen of the moose were used as probiotics in neonatal lambs for 9 weeks, the acetate/propionate ratio decreased and the abundance of *Prevotella*, *Butyrivibrio*, and *Ruminococcus* increased [77]. Supplementation with *Saccharomyces cerevisiae* in the diet of lambs between 1 and 180 days of age promoted cellulolytic bacterial colonization in the rumen, decreased the rumen ammonia concentration, and increase the VFA concentration [51]. In addition to the above factors, different feeding patterns (nursing with mother versus artificial feeding milk replacer), culture conditions (grazing versus house feeding), ruminant species, and environment affect the colonization of rumen microorganisms [34].

## 4. Conclusions

In summary, the establishment of the rumen microbiota in early life is crucial for the improvement of rumen function and immune system during pre-weaned ruminants. Several studies have found that early nutritional interventions can manipulate the establishment of rumen microbiota. However, whether early nutritional interventions have a lasting effect on adult animal growth and phenotypes needs to be investigated further. Furthermore, there is still a lack of understanding of the mechanisms that regulate rumen microbiome-host interactions during rumen development, which limits the development of new and effective feed additives for ruminants.

## Figures and Tables

**Table 1:** Factors influencing the rumen microbiota.

Factor	Host	Treatment	Results	References
Age	Goats	Days 0, 14, 28, 42, and 56	*Prevotella*, *Treponema*, *Ruminococcus*, and the unclassified family *Prevotellaceae* increase with the age of the kids.	[10]
	Lambs	Day 0, 3, 10, 20, 30, 45, 60, and 120	The rumen bacterial community and its functions were all affected by the age of the lambs.	[9]
Diet	Dairy calves	Milk-fed; milk plus starter concentrate fed	Feeding solid feed promoted a greater diversity of bacterial taxa known to degrade readily fermentable carbohydrates in the rumen. Milk-fed groups exhibit bacterial communities dominated by taxa that can utilize milk nutrients.	[22]
	Lambs	Adding alfalfa based on concentrate	The unclassified *Lachnospiraceae*, *Treponema*, and unclassified *Ruminococcaceae* were increased in starter supplemented with alfalfa groups during pre-weaning periods.	[48]
	Lambs	Linseed oil was added to the lambs’ diet	The relative abundance of *Succinivibrionaceae* and *Veillonellaceae* was higher in groups fed linseed oil before weaning, and the short-term addition of linseed oil to the diet of lambs in early life had a lasting effect on the community composition of rumen bacteria.	[5]
Genetic	Cattle bison	Transfer of rumen contents from bison to cattle	Inoculation with bison rumen contents alters the bison cattle rumen microbiome and metabolism.	[49]
	Beef cattle	A total of 709 beef cattle from three breeds were raised under the same feedlot conditions	Some rumen microbial features are heritable and may be influenced by host genetics.	[50]
Probiotics	Lambs	*Saccharomyces cerevisiae* CNCM I-1077 and their cultures	Live yeast supplements have induced the establishment of the eukaryotic families *Trichostomatia*, *Neocallimastigaceae*, and *Fibrobacter succinogenes*.	[51]
Rumen fluid transplantation	Lambs	Inoculation of lambs with adult sheep fresh rumen fluid	Fresh rumen fluid inoculation promoted the colonization of lamb by bacteria such as *Succiniclasticum*, *Prevotella*, and *Proteobacteria* S24-7.	[52]
Plant extract	Rumen-cannulated sheep	A total of 25 mg of resveratrol was added to a 300 mg high-concentrate or high-forage diet.	It reduces the abundance of *Methanobrevibacter* in the rumen fluid of lambs and increases the abundance of *Prevotella* and *Desulfovibrio*.	[53]

## Data Availability

Not applicable.
